# Matrix glycoconjugate characterization in multispecies biofilms and bioaggregates from the environment by means of fluorescently-labeled lectins

**DOI:** 10.3389/fmicb.2022.940280

**Published:** 2022-08-08

**Authors:** Thomas R. Neu, Ute Kuhlicke

**Affiliations:** Helmholtz Centre for Environmental Research – UFZ, Magdeburg, Germany

**Keywords:** biofilm, bioaggregates, biofilm matrix, extracellular polymeric substances, lectin, glycoconjugate, confocal laser scanning microscopy

## Abstract

Environmental biofilms represent a complex mixture of different microorganisms. Their identity is usually analyzed by means of nucleic acid-based techniques. However, these biofilms are also composed of a highly complex extracellular matrix produced by the microbes within a particular biofilm system. The biochemical identity of this extracellular matrix remains in many cases an intractable part of biofilms and bioaggregates. Consequently, there is a need for an approach that will give access to the fully hydrated structure of the extracellular matrix or at least a major part of it. A crucial compound of the matrix identified as carbohydrate-based polymers represents major structural and functional constituents. These glycoconjugates can be characterized by using fluorescently-labeled lectins in combination with confocal laser scanning microscopy. The lectin approach is defined previously, as fluorescence lectin barcoding (FLBC) and fluorescence lectin-binding analysis (FLBA), where FLBC is equal to the screening of a particular sample with all the commercially available lectins and FLBA is the actual analysis of the matrix throughout an experiment with a selected panel of lectins. As the application of immune-based techniques in environmental biofilm systems is impossible, the lectin approach is currently the only option for probing lectin-specific glycoconjugates in complex biofilms and bioaggregates. From all the commercially available lectins tested, the lectins such as AAL, HAA, WGA, ConA, IAA, HPA, and LEA showed the highest binding efficiency. Furthermore, 20 of the overall lectins tested showed an intermediate signal intensity, nevertheless very useful for the assessment of matrix glycoconjugates. With the data compiled, we shall virtually shed more light on the dark matter of the extracellular matrix and their 3-dimensional distribution in environmental biofilm systems. The results will be helpful in future studies with a focus on the extracellular matrix glycoconjugates present in environmental microbial communities.

## Introduction

The extracellular matrix of microbial communities, such as biofilms and bioaggregates, represents an essential part of their structure and functionality. This self-produced matrix is a complex mixture of different biochemical constituents, such as polysaccharides, proteins, extracellular nucleic acids, amphiphilic compounds, and microbial-derived refractory compounds (Neu and Lawrence, 2017). Usually, a mixture of these constituents summarized as extracellular polymeric substances (EPSs) built the matrix. However, several recent publications used the term extracellular matrix (ECM). The pivotal significance of the matrix in a wider sense was discussed in several overviews with a focus on the following aspects: general matrix facts and issues (Flemming and Wingender, 2010), giving structure to the matrix (Hobley et al., 2015), sensing and signaling of the matrix (Steinberg and Kolodkin-Gal, 2015), the functionality of matrix constituents (Neu and Lawrence, 2017), and matrix function in a social context (Dragoš and Kovács, 2017).

Nevertheless, analyzing the seemingly intractable matrix remains a major challenge. There are several approaches to analyzing the matrix. First, establishing extraction strategies and subsequent biochemical analysis (D'Abzac et al., 2010; Sun et al., 2012; Zhang et al., 2012; Pellicer-Nacher et al., 2013; Loustau et al., 2018); second, new extraction methods (Felz et al., 2016; Pronk et al., 2017; Boleij et al., 2018; Wong et al., 2020); third, taking advantage of genomics and proteomics techniques to examine the extracellular space (Dumas et al., 2008; Paes Leme et al., 2008; Cao et al., 2011; Albertsen et al., 2013; Yu et al., 2016); and fourth, *in-situ* 3-dimensional visualization by confocal laser scanning microscopy (Neu and Lawrence, 2014; Schlafer and Meyer, 2017).

The continuous challenge in analyzing the hydrated matrix *in situ* by means of visual techniques lies in its biochemical heterogeneity and the absence of a general contrasting agent. Ideally, a single probe for the extracellular matrix would be very useful either as a direct stain or as a labeled probe. However, even for one of the major matrix components, e.g., polysaccharides as a prominent example, an overall fluorescence staining approach is not available. Consequently, a compromise had to be established by taking advantage of fluorescently-labeled lectins and their glycoconjugate specificity (Neu and Lawrence, 1999; Neu et al., 2001). The lectin approach is inevitable for environmental samples, as there is no alternative such as the production and application of antibodies. Although immune-based techniques are powerful, they may be only applicable to pure culture studies.

This manuscript represents a follow-up on the lectin screening results already published on several pure culture studies with biofilms and bioaggregates (Neu and Kuhlicke, 2017). At the time, we defined the actual lectin screening as fluorescence lectin barcoding (FLBC) and the subsequent lectin analysis as fluorescence lectin-binding analysis (FLBA). FLBC requires a whole list of commercially available lectins for testing a particular sample type. FLBA stands for the tailor-made employment of selected lectins in a defined experiment. In contrast to the previous publication, this manuscript presents lectin data compiled from several environmental studies. We grouped the various FLBC results into the following biofilm systems: (1) derived from rivers, (2) developed in biofilm reactors, (3) wastewater reactor granules, (4) marine samples, and (5) miscellaneous. The lectin data presented in form of a barcoding table may be useful for similar applications and should serve as a guideline for the selection of lectins in studies on glycoconjugates of multispecies and environmental biofilm systems.

## Materials and methods

### Samples examined

Many samples examined by fluorescence lectin barcoding originate from a range of collaborative projects having very different backgrounds and motivations (see Section Acknowledgments). Publication of major results from these projects included a few selected lectin image data sets. Nevertheless, the presentation of the original and extensive lectin screening data is missing. Details of sample origin and development compiled in form of a table with reference to the original articles are given in Table 1.

**Table 1:** List of habitats and origin of biofilm samples together with the reference of originally published data.

**Habitat**	**References**
**Rivers, creeks**
Westerhöfer creek, tufa	Zippel and Neu, 2011
Deinschwanger creek, tufa	Zippel and Neu, 2011
Chriesbach, column	Derlon et al., 2016
Chriesbach, flow cells	Desmond et al., 2018
Elbe, river snow	Luef et al., 2009
Danube, river snow	Luef et al., 2009
**Flow lanes, reactors**
WWTP, flow lane, low light	Zippel et al., 2007
WWTP, flow lane, high light	Zippel et al., 2007
RAR, Elbe river water	Staudt et al., 2003
RAR, Elbe river water, and glucose	Staudt et al., 2003
RAR, Elbe river water, and methanol	Staudt et al., 2003
Paper mill, white water, reactor	Milferstedt et al., 2012
Cooling tower, industry	http://dottoratobee.uniroma2.it/files/2018/03/Di-Gregorio.pdf
**Reactor granules**
Anaerobic granules, low salt	Gagliano et al., 2018
Anaerobic granules, high salt	Gagliano et al., 2018
Anaerobic, anammox granules, EPS glycoprotein	Boleij et al., 2018, 2020
Aerobic reactor, flocs and granules	Weissbrodt et al., 2013
Aerobic granules, WWTP and acetate, acid soluble EPS	Pronk et al., 2017
Aerobic granules, WWTP and seawater, sialic acids	de Graaff et al., 2019
Aerobic granules, hyaluronic acid-like/glycosaminoglycans	Felz et al., 2020
Aerobic/anaerobic granules, nonulosonic acids	Tomás-Martínez et al., 2021
**Marine**
Hypersaline mat, zone 3	Arp et al., 2012
Hypersaline mat, zone 6	Arp et al., 2012
Hypersaline mat, zone 12	Arp et al., 2012
North sea, marine snow	Bennke et al., 2013
**Miscellaneous**
Iron snow, lignite mining lake	Lu et al., 2013
Microbial mat, hot spring	Ward et al., 2006
Cave snotties	Karwautz et al., 2018

RAR, rotating annular reactor; WWTP, wastewater treatment plant.

### Lectins staining and screening

Commercially available lectins purchased from various suppliers, such as Sigma-Aldrich, EY Laboratories, Vector Laboratories, and Molecular Probes (Supplementary Table 1), had labels of green-emitting fluorochromes. They comprised fluorescein isothiocyanate (FITC), fluorescein, or Alexa Fluor 488. For lectin combinations, orange-/red-/far red-labeled lectins are possible, such as tetramethylrhodamine isothiocyanate (TRITC), Texas Red, Cy5, or various Alexa fluorochromes. Conjugation of unlabeled lectins with Alexa fluorochromes using a kit from Molecular Probes according to their protocol allows the attachment of any fluorochrome matching sample properties. The lectins purchased (1 mg/ml buffer) were divided into aliquots and kept at −20°C. This stock solution was diluted at 1:10 for fluorescence staining of the biofilm matrix. Additional details reported elsewhere give further information (Neu and Lawrence, 1999, 2014).

For staining a particular sample, one biofilm or aggregate needs incubation with one lectin. Thus, a screening using, e.g., 80 different lectins requires 80 subsamples. Lectin staining is straightforward and was described in detail previously (Neu and Lawrence, 1999). In brief, the hydrated sample is exposed to some droplets of lectin solution and incubated for 20 min in the dark. Washing off the unbound lectins several times results in increased contrast. The washing procedure requires matching liquids, e.g., filter-sterilized water, buffer, or a suitable medium (no complex carbohydrate-containing constituents). There are several options for removing unbound lectins according to sample properties and fragility (Neu and Lawrence, 2014). If paraformaldehyde (PFA) fixed samples are used, replacement of PFA with water or buffer is necessary. The overall procedure of lectin staining was critically examined in an earlier report (Neu et al., 2001).

### Sample mounting and assessment

The mounting of samples was according to their origin, properties, and appearance. Biofilms are usually grown on solid surfaces, e.g., plastic cut into pieces of a few cm^2^. One piece was glued into a small 5 cm petri dish using a silicone sealant. The biofilms were stained with the lectin and washed based on their stability using a variety of options (Neu and Lawrence, 2014). Then, the petri dish was flooded with water or buffer and examined with water-immersible (dipping) lenses. To avoid squeezing the structure of flocs, aggregates, or granules, CoverWell chambers with various spacers proved to be ideal for mounting. After staining and washing bioaggregates, examination of the chamber using water immersion lenses through a No. 1.5H coverslip gave the best results.

In the first step, sample assessment was visually in the epifluorescence mode to identify those with no binding or weak binding patterns (no signal or brownish-green signal). If samples showed a strong or intermediate binding (bright green signal), a reference data set recorded in confocal mode is rather helpful for later comparison of lectin staining patterns.

### Confocal laser scanning microscopy

In the course of our study, two confocal laser scanning microscopes were available: a TCS SP1 and a TCS SP5X both with an upright microscope (Leica Microsystems, Germany). The TCS SP1 system was equipped with traditional laser sources (argon 488 nm, DPSS 561 nm, and HeNe 633 nm), controlled by the LCS software version 2.61. The TCS SP5X system was equipped with a supercontinuum laser light source (470–670 nm) and controlled by the software LAS-AF version 2.4.1. For recording image data sets, usually, excitation at 488 nm and collection of the emission signal from 500 to 550 nm (FITC, fluorescein, and Alexa Fluor 488) became standard for green-emitting fluorochromes. In most cases, the following objective lenses were employed: (1) 25 × NA 0.95 VISIR Fluotar and 63 × NA 0.9 HCX APO both water immersible (biofilms mounted in Petri dishes without a coverslip) and (2) 25 × NA 0.95 VISIR Fluotar and 63 × NA 1.2 corr CS HCX PL APO both water immersion lenses (flocs, aggregates, and granules in CoverWell chambers with coverslip).

### Image recording and data presentation

For most of the data sets, the general settings of recording parameters comprised as follows: 8-bit data depth, format or pixel resolution 512 × 512 (TCS SP1) or 1,024 × 1,024 (TCS SP5X), scan speed medium (TCS SP1) or 400 Hz (TCS SP5X), step size 1 μm, no average, and no zoom. As a standardized procedure, all the data sets recorded by using the lookup table “glow over under” (GOU) assured an optimal signal-to-noise ratio. Further, the pixel intensities showed only a few saturated pixels and a background level just above zero. Hence, with GOU, the full dynamic range of the pixel intensities is used.

The results of the screening may be presented as a binary color pattern with only black (binding) and white (no binding) information. More information extracted from a heat map facilitates the differentiation of several binding efficiencies. For this purpose, the voltage settings of the photomultiplier tube (PMT) are available as a convenient measure. The following PMT voltage settings defined 400–600 as a strong signal, 600–800 as an intermediate signal, and 800–1,000 as a weak signal. Compilation of the barcoding patterns presented in form of a color-coded Excel sheet (Microsoft) allows the selection of potential lectins for a more detailed assessment. For the final digital image analysis, the software Imaris (Bitplane) for visualization, Huygens (SVI) for deconvolution, and Photoshop (Adobe) for the presentation were available.

## Results

### Epifluorescence microscopy

**Figure 1:**
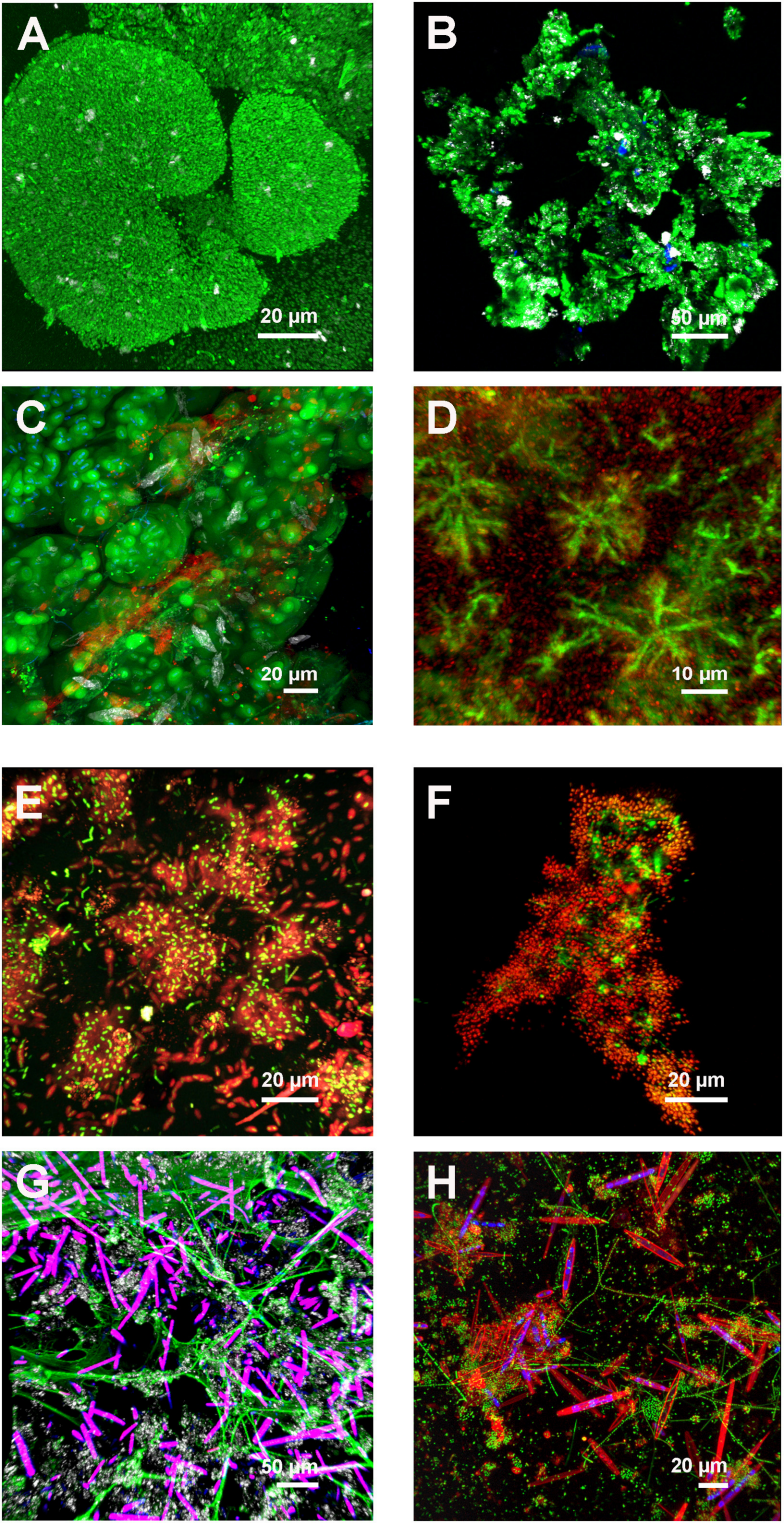
Examples of different lectin patterns from environmental biofilm and bioaggregate samples. The multichannel, 3-dimensional data sets recorded by CLSM and projected as maximum intensity projection (MIP) indicate a variety of lectin-specific glycoconjugates. **(A)** Reactor granule showing dense bacterial aggregates with cell surface RPA-lectin glycoconjugates and reflection signal. Color allocation: lectin—green, reflection–gray. Axial dimension: 42 μm, 86 optical sections. **(B)** Danube river snow aggregate showing ECA-lectin glycoconjugates. Color allocation: lectin–green, autofluorescence of chlorophyll–blue, and reflection–gray. Axial dimension: 59 μm, 60 optical sections. **(C)** Cave snotty sample with double lectin staining showing globular and filamentous lectin glycoconjugates. Color allocation: AAL-lectin–green, PNA-lectin–red, nucleic acid stain–blue, and reflection–gray. Axial dimension: 74 μm, 75 optical sections. **(D)** Biofilm reactor (white water) with star-like microcolonies linked by VVA-specific lectin glycoconjugates. Color allocation: lectin–green, nucleic acid stain–red. Axial dimension: 87 μm, 88 optical sections. **(E)** Rotating annular reactor biofilm developed from Elbe river water and fed with methanol. The young biofilm shows single cells and microcolonies covered with HPA-lectin glycoconjugates. Color allocation: nucleic acid stain–green, lectin–red. Axial dimension: 29 μm, 30 optical sections. **(F)** Anammox floc from a laboratory reactor showing PHA-E-lectin glycoconjugates. Color allocation: nucleic acid stain–green, lectin–red. Axial dimension: 34 μm, 18 optical sections. **(G)** Freshwater tufa sample showing net-like AAL-lectin glycoconjugates. Color allocation: lectin–green, autofluorescence overlay of phycobilin and chlorophyll–purple, and reflection–gray. Axial dimension: 118 μm, 60 optical sections. **(H)** Flow lane biofilm developed from river Elbe water showing diatoms with cell surface AAL-lectin signal and bacterial colonies embedded in AAL-lectin glycoconjugates. Color allocation: nucleic acid stain–green, lectin–red, and autofluorescence of chlorophyll–blue. Axial dimension: 50 μm, 51 optical sections.

For the characterization of the extracellular glycoconjugates in a new unknown biofilm or bioaggregate sample *via* FLBC, a screening with many different lectins was required as a basis for a more detailed examination. The commercially available lectins comprised about 70–80 different types. However, their supply changed depending on the availability of raw materials, biochemical isolation, and legal regulations. Hence, the number of available lectins required the same number of samples subsequently examined visually by epifluorescence microscopy. Samples stained with FITC-, fluorescein-, or Alexa Fluor 488-labeled lectins usually showed different shades of green. Weak binding patterns appeared as a brown-green signal, whereas a bright green signal indicated a strong binding signal. In any case, the signal should be matching a microbiological structure. In other words, one should consider the glycoconjugate structures in relation to microbial features (cells, microcolonies, films, flocs, aggregates, granules, and mats) potentially expected. If a positive lectin signal was identified in the epifluorescence mode visually, a sample data set in confocal mode by using the GOU lookup table was recorded for documentation. Any artificial fluorescence signals originating, e.g., from geometric minerals or detritus particles, were neglected. Examples from various screenings given in Figure 1 show different patterns of lectin-specific glycoconjugate signals from environmental samples.

### Binary barcoding

By means of an FLBC screening, often a Yes/No answer was only needed. For this purpose, a listing of the photomultiplier tube (PMT) setting in form of the voltage (sensitivity) for all the positively tested lectins may be enough. This identified the strongest binding pattern by showing the lowest PMT voltage setting. In addition, the transformation of the results into a simple binary barcoding pattern generated a graphic presentation based on a self-defined cutoff value meaning good binding or weak/no binding. There was, however, one aspect to consider, the nature of the lectin signal, meaning the structure of interest in view of the research focus. For example, the significance of glycoconjugates, e.g., on the cell surface, in microcolonies, throughout the extracellular biofilm matrix, and microbe–microbe or microbe–eukaryote interactions.

### Heat map barcoding

**Figure 2:**
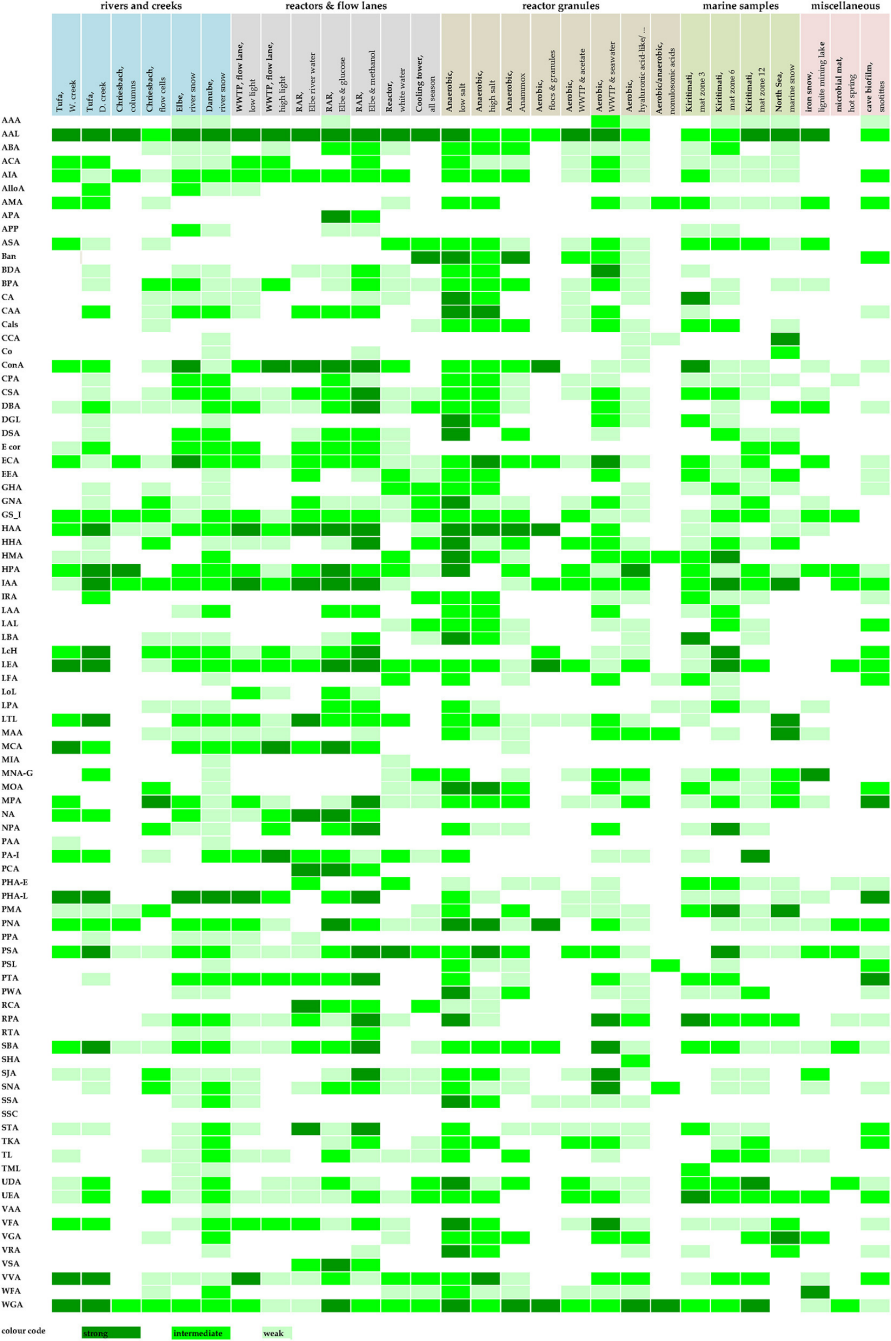
Detailed barcoding results of the lectin screening (FLBC) by means of a three color heat map. The signal intensities were color-coded equal to signal intensity: high signal (PMT voltage 400–600), intermediate signal (PMT voltage 600–800), and low signal (PMT voltage 800–1,000). Color coding: strong lectin binding–dark green, intermediate lectin binding–bright green, and low lectin binding–soft green. For abbreviations of lectins, see Supplementary Table 1.

More information revealed by grouping the results into several intensity clusters gave further details on lectin binding characteristics (Figure 2). The basis for this heat map was again the voltage setting of the PMT. From experience, we arbitrarily defined three binding efficiencies meaning three PMT ranges: 400–600, 600–800, and 800–1,000 V. As a result, the low sensitivity of the photomultiplier tube (400–600 V) indicated strong glycoconjugate signals, intermediate sensitivity of the photomultiplier tube (600–800 V) indicated good glycoconjugate signals, and high sensitivity of the photomultiplier tube (800–1,000 V) indicated weak glycoconjugate signals. Of note, the image data recorded using the lookup table GOU may look similar in intensity although recorded at different voltages. Furthermore, CLSM data recorded with a high voltage setting may contain some background noise. Therefore, it was necessary to use background subtraction, filtering, or deconvolution to improve the resolution of the data sets. In a second step, the FLBC data from the heat map provided a frame to perform a more detailed FLBA with selected lectins to follow glycoconjugate patterns throughout, e.g., a time experiment, interaction study, or any other biofilm experiment.

### Lectin information

The lectin applicability as revealed by FLBC should serve as a guideline for similar studies. This would avoid buying all the lectins commercially offered. Purchasing the full range of lectins, some of them with different fluorochrome labels would mean an investment in the range of 15–20 K calculated in €.

The signal intensity derived from image data lends itself to counting and shortlisting. In Table 2, those lectins are compiled showing a strong binding efficiency (dark green shading in Figure 2). The lectins listed according to their frequency of binding provided a shortlist of extremely valuable lectins. Table 2 also indicates the inhibitory carbohydrate if known in the literature. Consequently, Table 2 offers a powerful hint of which lectin to select for imaging lectin-specific glycoconjugates within a defined biofilm system. The most frequent lectin identified, AAL, has specificity for α-Fuc. The next best lectins, HAA and WGA, inhibited by β-GlcNAc, have also other specificities (see Table 2). The ConA is probably the most often applied lectin, as it is well characterized, has a bit broader specificity, is readily available, and is also one of the cheapest lectins. The lectin IAA is not well characterized but often showed a rather strong signal. Further down the list, the two lectins, namely HPA and LEA, are specific for α-GalNAc and β-GlcNAc, which were also very successfully applied (see Table 2).

**Table 2:** Shortlist of lectins bound with a strong binding efficiency (see dark green shading in Figure 2).

**Lectin**	**Specificity**
AAL	α-Fuc
HAA	α-GlcNAc, α-GalNAc
WGA	β-GlcNAc, sialic acid
ConA	α-Man, α-Glc, α-GlcNAc
IAA	n. d.
HPA	α-GalNAc
LEA	β-GlcNAc

Lectins are listed according to their binding frequency.

n. d.—lectin specificity not determined.

The lectins compiled in Table 3 with intermediate binding efficiency are still very useful for the examination of environmental biofilm systems for their lectin-specific glycoconjugates. The top scorer in this list, GS-I, has specificity for α-Gal and α-GalcNAc. The second lectin, AIA, has again specificity for α-Gal. Interestingly, the next four lectins in the list (LEA, IAA, WGA, and HPA) are also present, as shown in Table 2. This implies that they should be in focus if defining a selection of lectins. The lectins selected either applied as an individual lectin to a single biofilm or as a combination of lectins with different labels applied to one biofilm reveal additional information. The lectins further down the list show a variety of binding specificities and represent valuable probes too. Although some of the lectins might be similar in binding specificity according to the supplier's datasheet, they may show useful signals as these lectins may bind differentially.

**Table 3:** Shortlist of lectins bound with an intermediate binding efficiency (see bright green shading in Figure 2).

**Lectin**	**Specificity**
GS-I	α-Gal, α-GalcNAc
AIA	β-Gal
LEA	β-GlcNAc
IAA	n.d.
WGA	β-GlcNAc, sialic acid
HPA	α-GalNAc
UEA	α-Fuc, β-GlcNAc
ECA	α-Gal, β-Gal, α-GalNAc, β-GalNAc
PSA	α-Man, α-Glc, α-GlcNAc
ASA	α-Man
SBA	α-GalNAc, β-GalNAc
PNA	β-Gal
VVA	α-Man; α-GalNAc
AMA	n. d.
CSA	β-Gal
MPA	α-Gal, α-GalNAc
PA-I	Gal
PTA	β-Gal, α-GalNAc, β-GalNAc
UDA	β-GlcNAc
VFA	α-Man, α-Glc, α-GlcNAc

Lectins listed according to their binding frequency.

n. d.—lectin specificity not determined.

The results of Figure 2 can also be evaluated with respect to the habitat and sample origin. Four samples showed a strong binding efficiency with more than 10 lectins, with the “anaerobic reactor–low salt” binding 21 lectins. Four samples showed an intermediate binding efficiency with more than 30 lectins, again with the “anaerobic reactor–low salt” binding of 43 lectins. In general, with all the sample types tested, there were usually enough lectins to select for a more detailed FLBA study. If there are multiple options to choose from, it will be logical either to consider the specificity of the lectins or to look at the lectin signal pattern of interest. By this means, the establishment of a solid base for selecting a panel of useful lectins for a detailed FLBA investigation is feasible.

In view of a possible relationship between lectin binding vs. nonlectin binding and sample type, no clear pattern could be established. The reason might be the different origins of the samples (e.g., rivers and creeks) and the selective enrichment of a specific and differential microbial community (e.g., reactors, flow lanes, and reactor granules).

## Discussion

Visualization of the intact matrix in biofilm systems remains a challenge. Nevertheless, there are attempts to contrast the overall matrix. One approach based on reflection raises some issues and is not established in the field (Swearingen et al., 2016). Another approach, although promoted as a fluorescent matrix stain, the so-called “FilmTracer SYPRO Ruby biofilm matrix stain”, remains questionable. In fact, SYPRO Ruby as a general protein-specific fluorochrome will stain all the proteins, including the ones at the cell surface. In the case of polysaccharides as a major matrix polymer, there is no fluorochrome available that binds to all the types of polysaccharides. One fluorochrome sometimes used for matrix visualization is calcofluor white M2R. However, it will detect only one type of polysaccharides having a defined linkage type (β1 → 3, β1 → 4) as, for example, in cellulose (Rasconi et al., 2009). Furthermore, the option of using antibodies, as, for example, in pure culture studies, is not applicable in environmental samples. Consequently, the lectin approach applied to environmental microbiological samples, such as biofilms and bioaggregates, currently represents the only method to detect different matrix glycoconjugates. FLBC and FLBA in combination allow glycoconjugate imaging in hydrated samples, in 3-dimensions and *in situ*. The lectins selected for FLBA can be combined with other fluorochromes as contrasting agents, e.g., for staining cell distribution with nucleic acid-specific fluorochromes. A smart combination of different staining and imaging approaches will allow visualization of a complex 3-dimensional biofilm landscape in multiple channels (Neu and Lawrence, 2015; Lawrence et al., 2016, 2018).

It is important to be aware that if applying lectins, the direction of their binding specificity is not only toward polysaccharides but also to glycoconjugates in general. Glycoconjugates in microbial communities are present in form of glycolipids, glycoproteins, and polysaccharides. Hence, staining biofilm systems with a lectin will reveal different structural features. These may include (1) microbial cell surfaces such as capsules or sheaths (Zippel and Neu, 2011), (2) microbial footprints or holdfasts (Neu and Marshall, 1991; Neu, 1992; Baumgartner et al., 2016), (3) matrix structures within microcolonies (Lawrence et al., 2007, 2016), (4) microbe–microbe or microbe–eukaryote interactions (Kline et al., 2009; Bennke et al., 2013; Ielasi et al., 2016), and (5) overall matrix of the extracellular space (Staudt et al., 2004; Neu et al., 2005). Consequently, one should be aware of the biochemical and structural diversity to which the fluorochrome-labeled lectin may bind.

Lectin application is possible as individual probes and in combination. As indicated, many lectins are available with green-emitting fluorochromes. Other labels comprise orange or red and far-red emitting dyes. If lectins carrying these fluorochromes are not available, there are staining kits, which allow conjugation of any fluorescent color, e.g., with various Alexa dyes. This may be important if there are sample properties occupying a spectral window (e.g., in the far-red by chlorophyll A) or if other fluorochromes are added in combination (e.g., in the green part of the emission spectrum such as SYTO 9). Nevertheless, there is one option of separating multiple green fluorochromes based on the fluorescence lifetime. By means of fluorescence lifetime imaging microscopy (FLIM), separation of fluorochrome emitting in the same region is feasible, if they show a different lifetime. The FLIM technique, however, requires additional hardware and software. Although the FLIM option can be attached to a confocal laser scanning microscopy, it was hardly used in studies of microbial communities. Some examples with a focus on biofilm systems in combination with two-photon laser scanning microscopy are measuring pH in biofilms (Vroom et al., 1999), examination of bacterial activity (Walczysko et al., 2008), and microbial behavior under high pressure (Bourges et al., 2020). To the best of our knowledge, there is no report of FLIM for imaging the complexity of biofilm matrix constituents. In any case, if two or more lectins are applied, one has to make sure that they do not recognize each other forming a precipitate. Pairwise testing of two lectins with different fluorochromes on a microscope slide will easily show potential precipitates (Neu and Kuhlicke, 2017). Thus, a report on the application of a mixture with 20 different lectins has to be looked at critically (Fanesi et al., 2019).

As the lectin information collected by CLSM ends up as a digital data set, it is amenable to quantification. After thresholding, the pixel (2-dimensional) or voxel (3-dimensional) information may serve as a measure for glycoconjugate production. However, due to the many parameters to be controlled during CLSM, this will be only semiquantitative (Pawley, 2000). Nevertheless, there is a very recent tutorial for quantitative confocal microscopy with guidance to use the CLSM appropriately (Jonkman et al., 2020). Yet another aspect often raised applies to control and inhibition experiments. This issue already discussed reveals differential results, which need evaluation and control with respect to lectin binding characteristics (Zippel and Neu, 2011; Neu and Kuhlicke, 2017).

In the first manuscript on FLBC with pure cultures, the advantages and disadvantages have been elaborated (Neu and Kuhlicke, 2017). A positive aspect is the commercial availability of lectins, whereas a negative aspect may be the limited variation of their specificity. New specificities explored by means of the so-called carbohydrate-binding modules (CBMs) might offer additional probes (Nguyen et al., 2014; Ojima et al., 2015). Other candidates are lectins derived from microbial pili or fimbriae. Clearly, there is a need to have more lectins specific for some of the rare carbohydrates produced by microorganisms and lectins binding to the unique linkages between different microbial monosaccharides. This aspect was addressed in a statistical analysis of the Bacterial Carbohydrate Structure Database (Herget et al., 2008). Furthermore, the potential of lectin microarrays was discussed, as it will allow high-throughput analysis of many samples (Campanero-Rhodes et al., 2020), although this advantage was proven for only pure cultures (Yasuda et al., 2011).

## Conclusion

Fluorescence lectin barcoding constitutes a useful basis for shortlisting many lectins to perform a more detailed fluorescence lectin-binding analysis (FLBA). Thereby, assessment of glycoconjugate distribution and glycoconjugate typing becomes possible (Lawrence et al., 2007, 2016). The lectin approach combined with FISH or CARD-FISH enables the allocation of glycoconjugates to phylogenetic groups of bacteria (Böckelmann et al., 2002; Bennke et al., 2013). In addition to the previous manuscript with a focus on pure culture biofilms (Neu and Kuhlicke, 2017), there is a further important aspect concerning complex environmental biofilm systems. The key point and advantage of the lectin approach, including FLBC and FLBA, lies in its unique feature for detecting diverse glycoconjugates and its direct applicability on hydrated biofilm and bioaggregate samples. This aspect is crucial; as for environmental samples, it is impossible to apply immune techniques, e.g., producing antibodies against the vast diversity of potential matrix glycoconjugates. In terms of the lectin approach, new specificities are needed either by searching for new lectins or looking for similar proteins such as carbohydrate-binding modules as discussed previously (Neu and Kuhlicke, 2017). Finally, there is still a need for a fluorescent carbohydrate stain, which will allow contrasting the overall polysaccharide matrix in biofilm systems. Very likely, multiple strategies are necessary to address the large variety of polysaccharides, carbohydrate linkages, and the partly unique and exotic glycoconjugates present in microbial communities. All of these strategies should ideally match the techniques employed for *in-situ* visualization and analysis of other major matrix compounds, such as extracellular proteins and extracellular nucleic acids, amphiphilic compounds, and microbial-derived refractory constituents.

## Data availability statement

The original contributions presented in the study are included in the article/Supplementary material, further inquiries can be directed to the corresponding author.

## Author contributions

TN and UK worked at the confocal laser scanning microscopy analyzing the samples. UK handled the huge number of data sets recorded and compiled the lectin table/figure of the article. TN wrote the manuscript. All authors contributed to the article and approved the submitted version.

## Funding

TN and UK are thankful for the continuous financial support from the Helmholtz Centre for Environmental Research—UFZ in Leipzig over many years of laser scanning microscopy.

## Conflict of interest

The authors declare that the research was conducted in the absence of any commercial or financial relationships that could be construed as a potential conflict of interest.

## Publisher's note

All claims expressed in this article are solely those of the authors and do not necessarily represent those of their affiliated organizations, or those of the publisher, the editors and the reviewers. Any product that may be evaluated in this article, or claim that may be made by its manufacturer, is not guaranteed or endorsed by the publisher.

## References

[B1] Albertsen M. Stensballe A. Nielsen K. L. Nielsen P.-H. (2013). Digging into the extracellular matrix of a complex microbial community using a combined metagenomic and metaproteomic approach. Water Sci. Technol. 67, 1650–1656. 10.2166/wst.2013.030 23552257

[B2] Arp G. Helms G. Karlinska K. Schumann G. Reimer A. Reitner J. . (2012). Photosynthesis versus Exopolymer degradation in the formation of microbialites on the atoll of Kiritimati, Republic of Kiribati, Central Pacific. Geomicrobiol. J. 29, 29–65. 10.1080/01490451.2010.521436

[B3] Baumgartner M. Neu T. R. Blom J. F. Pernthaler J. (2016). Protistan predation interferes with bacterial long-term adaptation to substrate restriction by selecting for defence morphotypes. J. Evol. Biol. 29, 2297–2310. 10.1111/jeb.12957 27488245

[B4] Bennke C. M. Neu T. R. Fuchs B. M. Amann R. (2013). Mapping glycoconjugate-mediated interactions of marine Bacteroidetes with diatoms. Syst. Appl. Microbiol. 36, 417–425. 10.1016/j.syapm.2013.05.002 23809997

[B5] Böckelmann U. Manz W. Neu T. R. Szewzyk U. (2002). A new combined technique of fluorescent *in situ* hybridization and lectin-binding-analysis (FISH-LBA) for the investigation of lotic microbial aggregates. J. Microbiol. Methods 49, 75–87. 10.1016/S0167-7012(01)00354-2 11777585

[B6] Boleij M. Kleikamp H. Pabst M. Neu T. R. van Loosdrecht M. C. M. Lin Y. (2020). Decorating the anammox house: sialic acids and sulfated glycosaminoglycans in the extracellular polymeric substances of anammox granular sludge. Environ. Sci. Technol. 54, 5218–5226. 10.1021/acs.est.9b07207 32227885 PMC7181257

[B7] Boleij M. Pabst M. Neu T. R. van Loosdrecht M. C. M. Lin Y. (2018). Identification of glycoproteins isolated from extracellular polymeric substances of full-scale anammox granular sludge. Environ. Sci. Technol. 52, 13127–13135. 10.1021/acs.est.8b03180 30335377 PMC6256349

[B8] Bourges A. C. Lazarev A. Declerck N. Rogers K. L. Royer C. A. (2020). Quantitative high-resolution imaging of live microbial cells at high hydrostatic pressure. Biophys. J. 118, 2670–2679. 10.1016/j.bpj.2020.04.017 32402241 PMC7264842

[B9] Campanero-Rhodes M. A. Palma A. S. Menéndez M. Solís D. (2020). Microarray strategies for exploring bacterial surface glycans and their interactions with glycan-binding proteins. Front. Microbiol. 10, 2909. 10.3389/fmicb.2019.02909 32010066 PMC6972965

[B10] Cao B. Shi L. Brown R. N. Xiong Y. Fredrickson J. K. Romine M. F. . (2011). Extracellular polymeric substances from *Shewanella sp*. HRCR-1 biofilms: characterization by infrared spectroscopy and proteomics. Environ. Microbiol. 13, 1018–1031. 10.1111/j.1462-2920.2010.02407.x 21251176

[B11] D'Abzac P. Bordas F. van Hullebusch E. Lens P. N. L. Guibaud G. (2010). Extraction of extracellular polymeric substances (EPS) from anaerobic granular sludge: comparison of chemical and physical extraction protocols. Appl. Microbiol. Biotechnol. 85, 1589–1599. 10.1007/s00253-009-2288-x 19862516

[B12] de Graaff D. R. Felz S. Neu T. R. Pronk M. van Loosdrecht M. C. M. Lin Y. (2019). Sialic acids in the extracellular polymeric substances of seawater-adapted aerobic granular sludge. Water Res. 155, 343–351. 10.1016/j.watres.2019.02.040 30852321

[B13] Derlon N. Grütter A. Brandenberger F. Sutter A. Kuhlicke U. Neu T. R. . (2016). The composition and compression of biofilms developed on ultrafiltration membranes determine hydraulic biofilm resistance. Water Res. 102, 63–72. 10.1016/j.watres.2016.06.019 27318448

[B14] Desmond P. Best J. P. Morgenroth E. Derlon N. (2018). Linking composition of extracellular polymeric substances (EPS) to the physical structure and hydraulic resistance of membrane biofilms. Water Res. 132, 211–221. 10.1016/j.watres.2017.12.058 29331909

[B15] Dragoš A. Kovács Á. T. (2017). The peculiar functions of the bacterial extracellular matrix. Trends Microbiol. 25, 257–266. 10.1016/j.tim.2016.12.010 28089324

[B16] Dumas E. Meunier B. Berdague J.-L. Chambon C. Desvaux M. Hebraud M. (2008). Comparative analysis of extracellular and intracellular proteomes of *Listeria monocytogenes* strains reveals a correlation between protein expression and serovar. Appl. Environ. Microbiol. 74, 7399–7409. 10.1128/AEM.00594-08 18836007 PMC2592921

[B17] Fanesi A. Paule A. Bernard O. Briandet R. Lopes F. (2019). The architecture of monospecific microalgae biofilms. Microorganisms 7, 352. 10.3390/microorganisms7090352 31540235 PMC6780892

[B18] Felz S. Al-Zuhairy S. Aarstad O. A. van Loosdrecht M. C. M. Lin Y. M. (2016). Extraction of structural extracellular polymeric substances from aerobic granular sludge. JoVE 115, e54534. 10.3791/54534 27768085 PMC5092066

[B19] Felz S. Neu T. R. van Loosdrecht M. C. M. Lin Y. (2020). Aerobic granular sludge contains Hyaluronic acid-like and sulfated glycosaminoglycans-like polymers. Water Res. 169, 115291. 10.1016/j.watres.2019.115291 31734393

[B20] Flemming H.-C. Wingender J. (2010). The biofilm matrix. Nat. Rev. Micro. 8, 623–633. 10.1038/nrmicro2415 20676145

[B21] Gagliano M. C. Neu T. R. Kuhlicke U. Sudmalis D. Temmink H. Plugge C. M. (2018). EPS glycoconjugate profiles shift as adaptive response in anaerobic microbial granulation at high salinity. Front. Microbiol. 9, 1423. 10.3389/fmicb.2018.01423 30013532 PMC6036115

[B22] Herget S. Toukach P. V. Ranzinger R. Hull W. E. Knirel Y. A. von der Lieth C.-W. (2008). Statistical analysis of the Bacterial Carbohydrate Structure Data Base (BCSDB): characteristics and diversity of bacterial carbohydrates in comparison with mammalian glycans. BMC Struct. Biol. 8, 35. 10.1186/1472-6807-8-35 18694500 PMC2543016

[B23] Hobley L. Harkins C. MacPhee C. E. Stanley-Wall N. R. (2015). Giving structure to the biofilm matrix: an overview of individual strategies and emerging common themes. FEMS Microbiol. Rev. 39, 649–669. 10.1093/femsre/fuv015 25907113 PMC4551309

[B24] Ielasi F. S. Alioscha-Perez M. Donohue D. Claes S. Sahli H. Schols D. . (2016). Lectin-glycan interaction network-based identification of host receptors of microbial pathogenic adhesins. mBio 7, e00584. 10.1128/mBio.00584-16 27406561 PMC4958244

[B25] Jonkman J. Brown C. M. Wright G. D. Anderson K. I. North A. J. (2020). Tutorial: guidance for quantitative confocal microscopy. Nature Protocols 15, 1585–1611. 10.1038/s41596-020-0313-9 32235926

[B26] Karwautz C. Kus G. Stöckl M. Neu T. R. Lueders T. (2018). Microbial megacities fueled by methane oxidation in a mineral spring cave. ISME J. 12, 87–100. 10.1038/ismej.2017.146 28949325 PMC5739006

[B27] Kline K. A. Fälker S. Dahlberg S. Normark S. Henriques-Normark B. (2009). Bacterial adhesins in host-microbe interactions. Cell Host Microbe 5, 580–592. 10.1016/j.chom.2009.05.011 19527885

[B28] Lawrence J. R. Swerhone G. D. W. Kuhlicke U. Neu T. R. (2007). *In situ* evidence for microdomains in the polymer matrix of bacterial microcolonies. Can. J. Microbiol. 53, 450–458. 10.1139/W06-146 17538657

[B29] Lawrence J. R. Swerhone G. D. W. Kuhlicke U. Neu T. R. (2016). *In situ* evidence for metabolic and chemical microdomains in the structured polymer matrix of bacterial microcolonies. FEMS Microbiol. Ecol. 93, fiw183. 10.1093/femsec/fiw183 27562775

[B30] Lawrence J. R. Winkler M. Neu T. R. (2018). Multi-parameter laser imaging reveals complex microscale biofilm matrix in a thick (4,000 μm) aerobic methanol oxidizing community. Front. Microbiol. 9, e02186. 10.3389/fmicb.2018.02186 30333795 PMC6176653

[B31] Loustau E. Rols J.-L. Leflaive J. Marcato-Romain C.-E. Girbal-Neuhauser E. (2018). Comparison of extraction methods for the characterization of extracellular polymeric substances from aggregates of three biofilm-forming phototrophic microorganisms. Can. J. Microbiol. 64, 887–899. 10.1139/cjm-2018-0182 30011379

[B32] Lu S. Chourey K. Reiche M. Nietzsche S. Shah M. B. Neu T. R. . (2013). Insights into the structure and metabolic function of microbes that shape pelagic iron-rich aggregates (iron snow). Appl. Environ. Microbiol. 79, 4272–4281. 10.1128/AEM.00467-13 23645202 PMC3697501

[B33] Luef B. Neu T. R. Zweimüller I. Peduzzi P. (2009). Structure and composition of aggregates in two large European rivers, based on confocal laser scanning microscopy and image and statistical analysis. Appl. Environ. Microbiol. 75, 5952–5962. 10.1128/AEM.00186-09 19633114 PMC2747869

[B34] Milferstedt K. Godon J. J. Escudié R. Prasse S. Neyret C. Bernet N. (2012). Heterogeneity and spatial distribution of bacterial background contamination in pulp and process water of a paper mill. J. Indust. Microbiol. Biotechnol. 39, 1751–1759. 10.1007/s10295-012-1196-8 23007958

[B35] Neu T. Kuhlicke U. (2017). Fluorescence lectin bar-coding of glycoconjugates in the extracellular matrix of biofilm and bioaggregate forming microorganisms. Microorganisms 5, 5. 10.3390/microorganisms5010005 28208623 PMC5374382

[B36] Neu T. R. (1992). Microbial “footprints” and the general ability of microorganisms to label interfaces. Can. J. Microbiol. 38, 1005–1008. 10.1139/m92-165

[B37] Neu T. R. Lawrence J. R. (1999). Lectin-binding-analysis in biofilm systems. Methods Enzymol. 310, 145–152. 10.1016/S0076-6879(99)10012-0 10547788

[B38] Neu T. R. Lawrence J. R. (2014). “Advanced techniques for *in situ* analysis of the biofilm matrix (structure, composition, dynamics) by means of laser scanning microscopy,” in Microbial Biofilms - Methods and Protocols. Methods in Molecular Biology, eds G. Donelli (New York, NY: Springer), 43–64. 10.1007/978-1-4939-0467-9_4 24664825

[B39] Neu T. R. Lawrence J. R. (2015). Innovative techniques, sensors, and approaches for imaging biofilms at different scales. Trends Microbiol. 23, 233–242. 10.1016/j.tim.2014.12.010 25623967

[B40] Neu T. R. Lawrence J. R. (2017). “The extracellular matrix - an intractable part of biofilm systems,” in The Perfect Slime, eds H.-C. Flemming, T. R. Neu, and J. Wingender (London: IWA Publishing).

[B41] Neu T. R. Marshall K. C. (1991). Microbial “footprints” - a new approach to adhesive polymers. Biofouling 3, 101–112. 10.1080/08927019109378166

[B42] Neu T. R. Swerhone G. D. W. Böckelmann U. Lawrence J. R. (2005). Effect of CNP on composition and structure of lotic biofilms as detected with lectin-specific glycoconjugates. Aquatic Microb. Ecol. 38, 283–294. 10.3354/ame038283

[B43] Neu T. R. Swerhone G. D. W. Lawrence J. R. (2001). Assessment of lectin-binding analysis for *in situ* detection of glycoconjugates in biofilm systems. Microbiology 147, 299–313. 10.1099/00221287-147-2-299 11158347

[B44] Nguyen M. H. Ojima Y. Sakka M. Sakka K. Taya M. (2014). Probing of exopolysaccharides with green fluorescence protein-labeled carbohydrate-binding module in Escherichia coli biofilms and flocs induced by bcsB overexpression. J. Biosci. Bioeng. 118, 400–405. 10.1016/j.jbiosc.2014.03.005 24746734

[B45] Ojima Y. Suparman A. Nguyen M. H. Sakka M. Sakka K. Taya M. (2015). Exopolysaccharide assay in *Escherichia coli* microcolonies using a cleavable fusion protein of GFP-labeled carbohydrate-binding module. J. Microbiol. Methods 114, 75–77. 10.1016/j.mimet.2015.05.013 25978970

[B46] Paes Leme A. F. Bellato C. M. Bedi G. Del Bel Cury A. A. Koo H. Cury J. A. (2008). Effects of Sucrose on the extracellular matrix of plaque-like biofilm formed *in vivo*, studied by proteomic analysis. Caries Res. 42, 435–443. 10.1159/000159607 18832830 PMC2820338

[B47] Pawley J. B. (2000). The 39 steps: a cautionary tale of quantitative 3-d fluorescence microscopy. BioTechniques 28, 884–887. 10.2144/00285bt01 10818693

[B48] Pellicer-Nacher C. Domingo-Felez C. Mutlu A. G. Smets B. F. (2013). Critical assessment of extracellular polymeric substances extraction methods from mixed culture biomass. Water Res. 47, 5564–5574. 10.1016/j.watres.2013.06.026 23866135

[B49] Pronk M. Neu T. R. van Loosdrecht M. C. M. Lin Y. M. (2017). The acid soluble extracellular polymeric substance of aerobic granular sludge dominated by Defluviicoccus sp. Water Res. 122, 148–158. 10.1016/j.watres.2017.05.068 28599160

[B50] Rasconi S. Jobard M. Jouve L. Sime-Ngando T. (2009). Use of calcofluor white for detection, identification, and quantification of phytoplanktonic fungal parasites. Appl. Environ. Microbiol. 75, 2545–2553. 10.1128/AEM.02211-08 19233954 PMC2675195

[B51] Schlafer S. Meyer R. L. (2017). Confocal microscopy imaging of the biofilm matrix. J. Microbiol. Methods 138, 50–59. 10.1016/j.mimet.2016.03.002 26979645

[B52] Staudt C. Horn H. Hempel D. C. Neu T. R. (2003). “Screening of lectins for staining lectin-specific glycoconjugates in the EPS of biofilms,” in Biofilms in Medicine, Industry and Environmental Technology, eds P. Lens, A. P. Moran, T. Mahony, P. Stoodley, and V. O'Flaherty (London, UK: IWA Publishing), 308–327.

[B53] Staudt C. Horn H. Hempel D. C. Neu T. R. (2004). Volumetric measurements of bacterial cells and extracellular polymeric substance glycoconjugates in biofilms. Biotechnol. Bioeng. 88, 585–592. 10.1002/bit.20241 15470707

[B54] Steinberg N. Kolodkin-Gal I. (2015). The matrix reloaded: how sensing the extracellular matrix synchronizes bacterial communities. J. Bacteriol. 197, 2092–2103. 10.1128/JB.02516-14 25825428 PMC4455261

[B55] Sun M. Li W. W. Yu H. Q. Harada H. (2012). A novel integrated approach to quantitatively evaluate the efficiency of extracellular polymeric substances (EPS) extraction process. Appl. Microbiol. Biotechnol. 96, 1577–1585. 10.1007/s00253-012-4478-1 23064456

[B56] Swearingen M. C. Mehta A. Mehta A. Nistico L. Hill P. J. Falzarano A. R. . (2016). A novel technique using potassium permanganate and reflectance confocal microscopy to image biofilm extracellular polymeric matrix reveals non-eDNA networks in Pseudomonas aeruginosa biofilms. Pathogens Dis. 74, ftv104. 10.1093/femspd/ftv104 26536894 PMC4675839

[B57] Tomás-Martínez S. Kleikamp H. B. C. Neu T. R. Pabst M. Weissbrodt D. G. van Loosdrecht M. C. M. . (2021). Production of nonulosonic acids in the extracellular polymeric substances of “*Candidatus* Accumulibacter phosphatis”. Appl. Microbiol. Biotechnol. 105, 3327–3338. 10.1101/2020.11.02.365007 33791836 PMC8053191

[B58] Vroom J. M. Grauw d,. C. J. Gerritsen H. C. Bradshaw A. M. Marsh P. D. Watson G. K. . (1999). Depth penetration and detection of pH gradients in biofilms by two-photon excitation microscopy. Appl. Environ. Microbiol. 65, 3502–3511. 10.1128/AEM.65.8.3502-3511.1999 10427041 PMC91526

[B59] Walczysko P. Kuhlicke U. Knappe S. Cordes C. Neu T. R. (2008). *In situ* activity measurement of suspended and immobilized microbial communities by Fluorescence Lifetime Imaging (FLIM). Appl. Environ. Microbiol. 74, 294–299. 10.1128/AEM.01806-07 17981940 PMC2223234

[B60] Ward D. M. Bateson M. M. Ferris M. J. Kühl M. Wieland A. Koeppel A. . (2006). Cyanobacterial ecotypes in the microbial mat community of Mushroom Spring (Yellowstone National Park, Wyoming) as species-like units linking microbial community composition, structure and function. Philos. Trans. R. Soc. Biol. Sci. 361, 1997–2008. 10.1098/rstb.2006.1919 17028085 PMC1764927

[B61] Weissbrodt D. G. Neu T. R. Kuhlicke U. Rappaz Y. Holliger C. (2013). Assessment of bacterial and structural dynamics in aerobic granular biofilms. Front. Microbiol. 4, e00175. 10.3389/fmicb.2013.00175 23847600 PMC3707108

[B62] Wong L. L. Natarajan G. Boleij M. Thi S. S. Winnerdy F. R. Mugunthan S. . (2020). Extracellular protein isolation from the matrix of anammox biofilm using ionic liquid extraction. Appl. Microbiol. Biotechnol. 104, 3643–3654. 10.1007/s00253-020-10465-7 32095864

[B63] Yasuda E. Tateno H. Hirabarashi J. Iino T. Sako T. (2011). Lectin microarray reveals binding profiles of *Lactobacillus casei* strains in a comprehensive analysis of bacterial cell wall polysaccharides. Appl. Environ. Microbiol. 77, 4539–4546. 10.1128/AEM.00240-11 21602390 PMC3127709

[B64] Yu W. Chen Z. Shen L. Wang Y. Li Q. Yan S. . (2016). Proteomic profiling of Bacillus licheniformis reveals a stress response mechanism in the synthesis of extracellular polymeric flocculants. Biotechnol. Bioeng. 113, 797–806. 10.1002/bit.25838 26388297

[B65] Zhang L. Ren H. Ding L. (2012). Comparison of extracellular polymeric substances (EPS) extraction from two different activated sludges. Water Sci. Technol. 66, 1558–1564. 10.2166/wst.2012.295 22864444

[B66] Zippel B. Neu T. R. (2011). Characterization of glycoconjugates of extracellular polymeric substances in tufa-associated biofilms by using fluorescence lectin-binding analysis. Appl. Environ. Microbiol. 77, 506–516. 10.1128/AEM.01660-10 21097578 PMC3020524

[B67] Zippel B. Rijstenbil J. Neu T. R. (2007). A flow-lane incubator for studying freshwater and marine phototrophic biofilms. J. Microbiol. Methods 70, 336–345. 10.1016/j.mimet.2007.05.013 17590463

