# Supplementary material for: Matrix glycoconjugate characterization in multispecies biofilms and bioaggregates from the environment by means of fluorescently-labeled lectins
Source: Front Microbiol. 2022 Aug 8;13:940280. doi: 10.3389/fmicb.2022.940280 (PMC9395170; doi:10.3389/fmicb.2022.940280)
Supplement: Supplementary file 1 [file Table_1.docx]

**Supplementary Table 1:** Overview of lectins available, sorted alphabetically according to their three-letter code. Nearly all of them were employed for the individual screenings and bar coding. The lectins listed were purchased from Sigma, EY Laboratories, Vector Laboratories and Molecular Probes.

| **Nr.** | **Lectin** | | | **Fluo label** | **Inhibiting carbohydrate** | **Linkage type** |
| --- | --- | --- | --- | --- | --- | --- |
|  | **Latin name** | **Common name** | **3-letter code** |  |  |  |
| 1 | *Anguilla anguilla* | fresh water eel | AAA | FITC | α-Fuc |  |
| 2 | *Aleuria aurantia* | mushroom | AAL | Alexa488, FITC | α-Fuc | Fuc(α1,6)GlcNAc, Fuc(α1,3)GalNAc, Fuc(α1,2)Gal(β1,4)[Fuc(α1,3/4)]Gal(β1,4)GlcNAc |
| 3 | *Agaricus bisporus* | mushroom | ABA | FITC | α-Gal | Gal(β1,3)GalNAc |
| 4 | *Amaranthus caudatus* | Inka wheat | ACA | FITC |  | Gal(β1,3)GalNAc |
| 5 | *Artocarpus integrifolia* | jackfruit | AIA | FITC | β-Gal | Gal(β1,3) GalNAc |
| 6 | *Allomyrina dichotoma* | Japanese beetle | AlloA | FITC | β-Gal |  |
| 7 | *Arum maculatum* | lords and ladies tubers | AMA | FITC |  | Man |
| 8 | *Abrus precatorius* | Jequirity bean | APA | FITC | β-D-Gal, GalNAc |  |
| 9 | *Aegopodium podagraria* | ground elder | APP | FITC | α-GalNAc, β-GalNAc | not determined |
| 10 | *Allium sativum* | garlic | ASA | FITC | α-Man | D-Man(α1,3) |
| 11 | *Musa paradisiaca* | banana | Ban | Fluo | Glc, Man | (α 1,3) glucosyl- and mannosyl- residues |
| 12 | *Bryonia dioica* | white bryony | BDA | FITC | α-GalNAc, β-GalNAc | not determined |
| 13 | *Bauhinia purpurea* | camel's foot tree | BPA | FITC | α-GalNAc, β-GalNAc |  |
| 14 | *Colchicum autumnale* | autumn crocus | CA | FITC |  | not determined |
| 15 | *Caragana aborescens* | pea tree | CAA | FITC | α-Gal, β-Gal, α-GalNAc, β-GalNAc |  |
| 16 | *Calystega sepiem* | hedge bindweed | Calsepa | FITC |  | Man>Glc>Fuc>GlcNAc |
| 17 | *Cancer antennarius* | California crab | CCA | Alexa488 | sialic acid | 9-O-Ac-NeuAc>4-O-Ac-NeuAc |
| 18 | *Codium fragile* | green sea fingers | Co | Alexa488 | GalNac |  |
| 19 | *Canavalia ensiformis* | jack bean | ConA | FITC | α-Man, α-Glc, α-GlcNAc | branched Man |
| 20 | *Cicer arietinum* | chick pea | CPA | FITC |  | Not determined |
| 21 | *Cytisus scoparius* | Scotch broom | CSA | FITC | β-Gal | lactose |
| 22 | *Dolichos biflorus* | horse gram | DBA | FITC | α-GalNAc | GalNAc(α1,3)GalNAc |
| 23 | *Dioclea grandiflora* |  | DGL | FITC | Man, Glc |  |
| 24 | *Datura stramonium* | jimson weed | DSA | FITC | β-GlcNAc | GlcNAc(β1,4)GlcNAc oligomers, Gal(β1,4)GlcNAc |
| 25 | *Erythrina cristagalli* | cockspur coral tree | ECA | FITC | α-Gal, β-Gal, α-GalNAc, β-GalNAc | Gal(β1,4)GlcNAc |
| 26 | *Erythrina corallodendron* | coral tree | Ecor | Alexa488 | Gal, GalNAc |  |
| 27 | *Euonymus europaeus* | spindle tree | EEA | Fluo |  | Gal(α1,3)[Fuc(α1,2)]Gal |
| 28 | *Glechoma hederacea* | ground ivy | GHA | FITC | GalNAc |  |
| 29 | *Galanthus nivalis* | snowdrop | GNA | FITC | α-Man | Man(α1,3)Man |
| 30 | *Griffonia simplicifolia* |  | GS-I | FITC | α-Gal, α-GalcNAc |  |
| 31 | *Helix aspersa* | garden snail | HAA | FITC | α-GlcNAc, α-GalNAc | not determined |
| 32 | *Hippeastrum hybrid* | amaryllis | HHA | FITC | α-Man | Man(α1,3) Man(α1,3) Man(α1,3) Man(α1,2)Man |
| 33 | *Homarus americanus* | California lobster | HMA | Alexa488, FITC | Sialic Acid, α-Fuc, α-GalNAc |  |
| 34 | *Helix pomatia* | edible snail | HPA | FITC | α-GalNAc | not determined |
| 35 | *Iberis amara* | candy tuft | IAA | Alexa488 |  | not determined |
| 36 | *Iris hybrid* | dutch Iris | IRA | FITC |  |  |
| 37 | *Laburnum alpinum* | Scotch alburnum | LAA | FITC | β-GlcNAc | GlcNAc(β1,4)GlcNAc |
| 38 | *Laburnum anagyroides* | golden chain/rain | LAL | FITC | α-Me-L-Fuc | Fuc(α1,2)Gal(β1,4)Glu and Fuc(α1,2)Ga(β1,4)Glc(β1,6)GalNAc[(β1,3) Gal] |
| 39 | *Phaseolus lunatus* | lima bean | LBA | FITC | α-GalNAc | GalNAc(α1,3)[Fuc-(α1,2)]Gal |
| 40 | *Lens culinaris* | lentil | LcH | FITC | α-Man, α-Glc, α-GlcNAc | branched Man with α-Fuc as determinant |
| 41 | *Lycopersicon esculentum* | tomato | LEA | FITC | β-GlcNAc | GlcNAc(β1,4)GlcNAc oligomers |
| 42 | *Limax flavus* | garden slug | LFA | FITC | sialic acid |  |
| 43 | *Lathyrus ochrus* | Cyprus vetch | LoL | FITC | α-Man, α-Glc |  |
| 44 | *Limulus polyphemus* | horseshoe crab | LPA | FITC | sialic acid |  |
| 45 | *Tetragonolobus purpurea* | asparagus pea | LTL | FITC | α-Fuc |  |
| 46 | *Maackia amurensis* | maackia | MAA | FITC | sialic acid, Gal |  |
| 47 | *Momordica charantia* |  | MCA | FITC | Gal, GalNAc |  |
| 48 | *Mangifera indica* | mango | MIA | Alexa488 |  |  |
| 49 | *Morniga G* | black mulberry | MNA-G | FITC |  | Gal |
| 50 | *Marasmius oreades* | mushroom | MOA | FITC |  | Gal(α1,3)Gal, Gal(α1,3)Gal(β1,4)GlcNAc/Glc |
| 51 | *Maclura pomifera* | osage orange | MPA | FITC | α-Gal, α-GalNAc | Gal(β1,3)GalNAc |
| 52 | *Naja mosambica* | cobra | NA | FITC |  |  |
| 53 | *Narcissus pseudonarcissus* | daffodil | NPA | FITC |  | Not determined |
| 54 | *Perseau americana* | avocado | PAA | Alexa488 |  |  |
| 55 | *Pseudomonas aeruginosa* |  | PA-I | Alexa488 | Gal |  |
| 56 | *Phaseolus coccineus* |  | PCA | FITC |  |  |
| 57 | *Phaseolus vulgaris* | red kidney bean | PHA-E | FITC |  | Gal |
| 58 | *Phaseolus vulgaris* | red kidney bean | PHA-L | FITC |  | Gal, GlcNAc, Man |
| 59 | *Polygonatum multiflorum* | solomon`s seal | PMA | FITC |  | Man |
| 60 | *Arachis hypogaea* | peanut | PNA | FITC | β-Gal | Gal(β1,3)GalNAc |
| 61 | *Ptilota plumosa* | red marine algae | PPA | Alexa488 |  |  |
| 62 | *Pisum sativum* | garden pea | PSA | FITC | α-Man, α-Glc, α-GlcNAc | branched Man with α-Fuc as determinant |
| 63 | *Polyporus squamosus* | mushroom | PSL | FITC |  | Neu5Ac(α2,6)Gal(β1,4)GlcNAc |
| 64 | *Psophocarpus tetragonolobus* | winged bean | PTA | FITC | β-Gal, α-GalNAc, β-GalNAc | Gal |
| 65 | *Phytolacca americana* | pokeweed | PWA | FITC | β-GalNAc | GlcNAc(β1,4)GlcNAc oligomers and [Gal-(β1,4)lcNAc]_2_ |
| 66 | *Ricinus communis* |  | RCA | Fluo | Gal, GalNAc |  |
| 67 | *Robinia pseudoaccacia* | black locust | RPA | FITC |  | not determined |
| 68 | *Trifolium repens* | white clover | RTA | Alexa488 |  |  |
| 69 | *Glycine max* | soybean | SBA | FITC | α-GalNAc, β-GalNAc | GalNAc(α1,3)Gal |
| 70 | *Salvia hormonum* |  | SHA | Alexa488 | GalNAc |  |
| 71 | *Sophora japonica* | Japanese pagoda tree | SJA | FITC | β-GalNAc | GalNAc(β1,6)Gal |
| 72 | *Sambucus nigra* | elderberry | SNA | Fluo | β-Gal, sialic acid | (α2,6)Gal, GalNAc |
| 73 | *Salvia sclarea* |  | SSA | FITC | GalNAc |  |
| 74 | *Sarothamnus scoparius* |  | SSC | Alexa488 |  |  |
| 75 | *Solanum tuberosum* | potato | STA | FITC | β-GlcNAc | GlcNAc(β1,4)GlcNAc oligomers |
| 76 | *Trichosanthes kirilowii* | Chinese cucumber | TKA | FITC | β-Gal | lactose |
| 77 | *Tulipa sp* | tulip | TL | FITC | α-GalNAc, β-GalNAc | GalNAc, Gal, Fuc |
| 78 | *Tritrichomonas mobilensis* |  | TML | Alexa488 |  |  |
| 79 | *Urtica dioica* | stinging nettle | UDA | FITC | β-GlcNAc | not determined |
| 80 | *Ulex europaeus* | gorse or furze | UEA | FITC | α-Fuc, β-GlcNAc | L-Fuc(α-1,2)Gal(β1,4)-GlcNAc |
| 81 | *Viscum album* |  | VAA | FITC |  | β-D-Gal |
| 82 | *Vicia faba* | fava bean, broad bean | VFA | FITC | α-Man, α-Glc, α-GlcNAc | branched Man with α-Fuc as determinant |
| 83 | *Vicia graminea* |  | VGA | Alexa488, FITC |  | O-linked Gal(β1,3)GalNAc |
| 84 | *Vigna radiata* | mung bean | VRA | FITC | α-Gal |  |
| 85 | *Vicia sativa* |  | VSA | FITC | Glc, Man |  |
| 86 | *Vicia villosa* | hairy vetch | VVA | FITC | α-Man; α-GalNAc | Man, GalNAc(α1,3)Gal |
| 87 | *Wisteria floribunda* | japanese wisteria | WFA | FITC | α-GalNAc, β-GalNAc | GalNAc(α1,6)Gal |
| 88 | *Triticum vulgaris* | wheat | WGA | FITC | β-GlcNAc; Sialic acid | GlcNAc(β1,4)GlcNAc |
